# Transcatheter Arterial Embolization as a Treatment for Chronic Pain due to Temporomandibular Joint Osteoarthritis

**DOI:** 10.1007/s00270-025-04008-8

**Published:** 2025-03-24

**Authors:** Florian Nima Fleckenstein, Jan Voss, Christian Doll, Tazio Maleitzke, Tobias Winkler, Eberhard Siebert, Federico Collettini

**Affiliations:** 1 Department of Diagnostic and Interventional Radiology, Charité Universitätsmedizin Berlin, Corporate Member of Freie Universität Berlin and Humboldt-Universität zu Berlin, Berlin, Germany; 2 Berlin Institute of Health at Charité Universitätsmedizin, Berlin, Germany; 3 Department of Oral and Maxillofacial Surgery, Charité Universitätsmedizin Berlin, Corporate Member of Freie Universität Berlin and Humboldt-Universität zu Berlin, Berlin, Germany; 4 Center for Musculoskeletal Surgery, Charité Universitätsmedizin Berlin, Corporate Member of Freie Universität Berlin and Humboldt-Universität zu Berlin, Berlin, Germany; 5 Berlin Institute of Health at Charité Universitätsmedizin Berlin, Julius Wolff Institute, Berlin, Germany; 6 Department of Orthopedic Surgery, Trauma Orthopedic Research Copenhagen Hvidovre (TORCH), Copenhagen University Hospital - Amager and Hvidovre, Hvidovre, Denmark; 7 Department of Clinical Medicine, University of Copenhagen, Copenhagen, Denmark; 8 Institute of Neuroradiology, Charité Universitätsmedizin Berlin, Corporate Member of Freie Universität Berlin and Humboldt-Universität zu Berlin, Berlin, Germany; 9 Department of Radiology, Charité - Universitätsmedizin Berlin, Charité Campus Mitte (CCM),Charitéplatz 1, Berlin, 10117 Germany

**Keywords:** Temporomandibular Joint, Osteoarthritis, Interventional Radiology, Pain Management, Musculoskeletal Diseases

## Abstract

**Purpose:**

To assess the efficacy and safety of transarterial embolization (TAE) for the treatment of temporomandibular joint osteoarthritis (TMJ-OA)-related symptoms.

**Materials and Methods:**

Three female patients were referred to our center for TAE after conservative and surgical TMJ treatments failed. Six TAE procedures were performed with bilateral treatments spaced four weeks apart. Following CBCT with maximal magnification and narrow collimation to confirm correct positioning of the microcatheter, superselective TAE was performed using Imipenem/Cilastatin mixed with contrast medium. Technical success was defined by successful embolization of the target vessel. Outcome measures included Oral Health Impact Profile—Temporomandibular Joint (OHIP-TMD) and Numeric Rating Scale (NRS) at baseline, 4 weeks and at 3 months intervals.

**Results:**

TAE was technically successful in all six procedures. No adverse events were recorded. Clinical follow-up data after 3 months are available for all three patients, one patient reached the 6-months follow-up. OHIP-TMD scores decreased from 38 to 31, 45 to 39, and 45 to 28, respectively. NRS pain scores improved from 9 to 6, 10 to 7, and 9 to 5, respectively.

**Conclusion:**

TAE appears to be a feasible and safe minimally-invasive option for selected TMJ-OA patients with symptoms refractory to standard treatments. Further studies with larger cohorts and extended follow-up are warranted to confirm these preliminary findings.

**Graphical Abstract:**

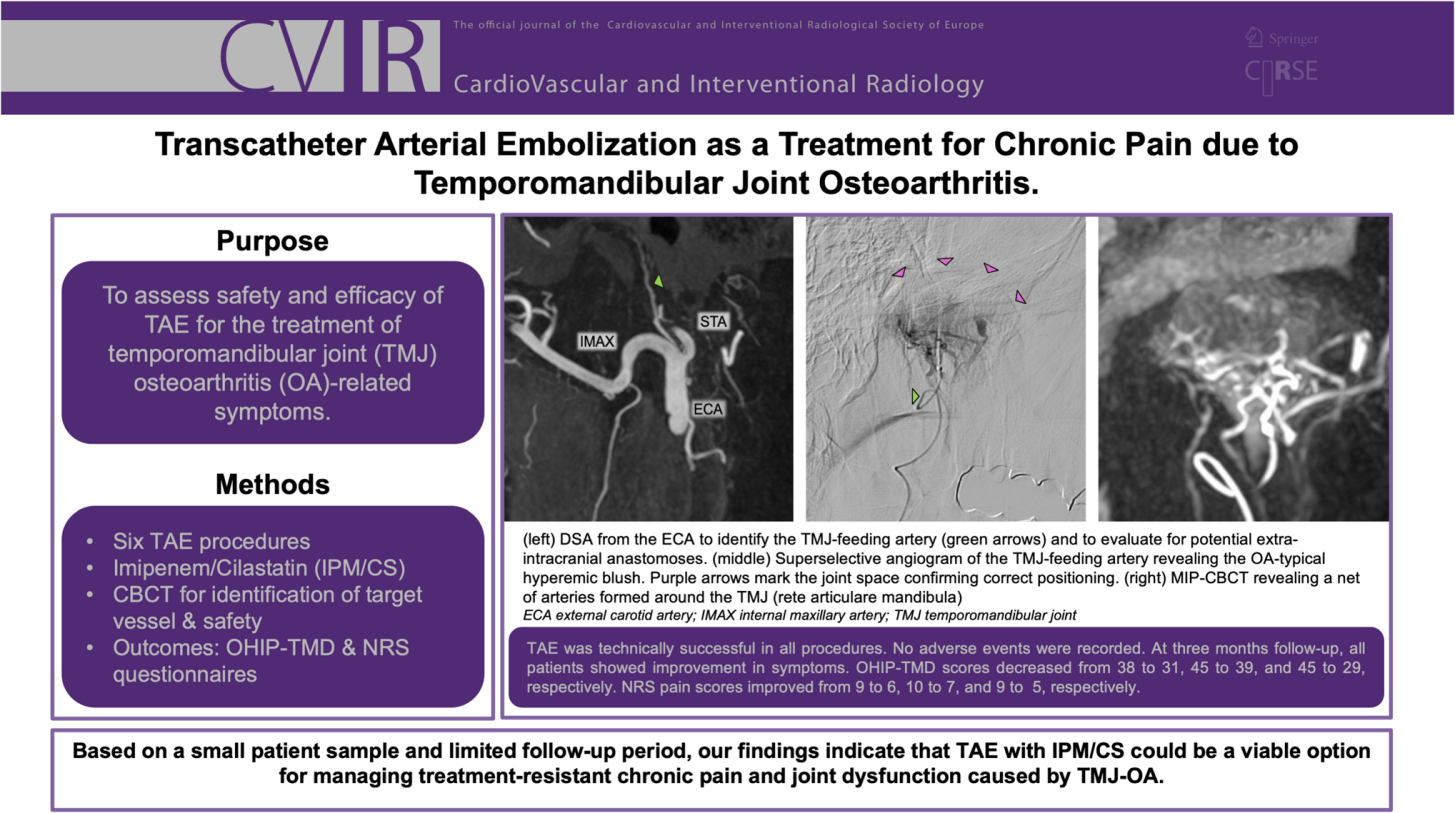

## Introduction

Temporomandibular joint osteoarthritis (TMJ-OA) is a debilitating condition characterized by progressive synovitis, cartilage destruction, and subchondral bone remodeling, leading to pain, limited jaw movement, and compromised quality of life [1]. Current treatments for TMJ-OA are mostly based on nonsurgical methods, including the use of analgesic and anti-inflammatory drugs, physical therapy, splint therapy, as well as minimally-invasive approaches such as joint cavity irrigation, and intra-articular injection of corticosteroids or hyaluronic acid [2]. Conventional therapies, including open surgery with replacement of the TMJ are generally associated with moderate success rates. For TMJ replacement, studies report success rates as low as 70%, accompanied by significant risk of nerve damage (44%) or the need for revision surgery due to infection (8%) [1, 3, 4]. There is hence a high clinical need for innovative and minimal-invasive therapies, able to alleviate symptoms and enhance joint function [5].

In recent years, transarterial embolization (TAE) has gained great attention for its potential to target the hypervascularity of the inflamed synovium and subchondral bone, which are critical contributors in the pathogenesis and to symptoms of osteoarthritis (OA) [6]. Recent research on TAE, has demonstrated its safety and effectiveness not only in large joints like the knee or shoulder, but also in smaller joints such as the trapeziometacarpal joint, as well as the distal and proximal interphalangeal joints [7–9]. The purpose of this short communication is to assess feasibility, safety, and technique of TAE applied to the TMJ for the treatment of OA-related symptoms.

## Materials and Methods

### Patients

This retrospective case series involved three consecutive patients who underwent TAE of the TMJ between December 2023 and October 2024. Patients were referred to us by oral and maxillofacial surgeons due to OA-related TMJ pain and dysfunctionality. Before treatment, written informed consent was obtained. All patients included in this study had previously undergone conservative treatment, including splint and physical therapy. The study was approved by our institutional review board (EA2/096/24).

### Patient Work-Up and Interventional Technique

**Fig. 1:**
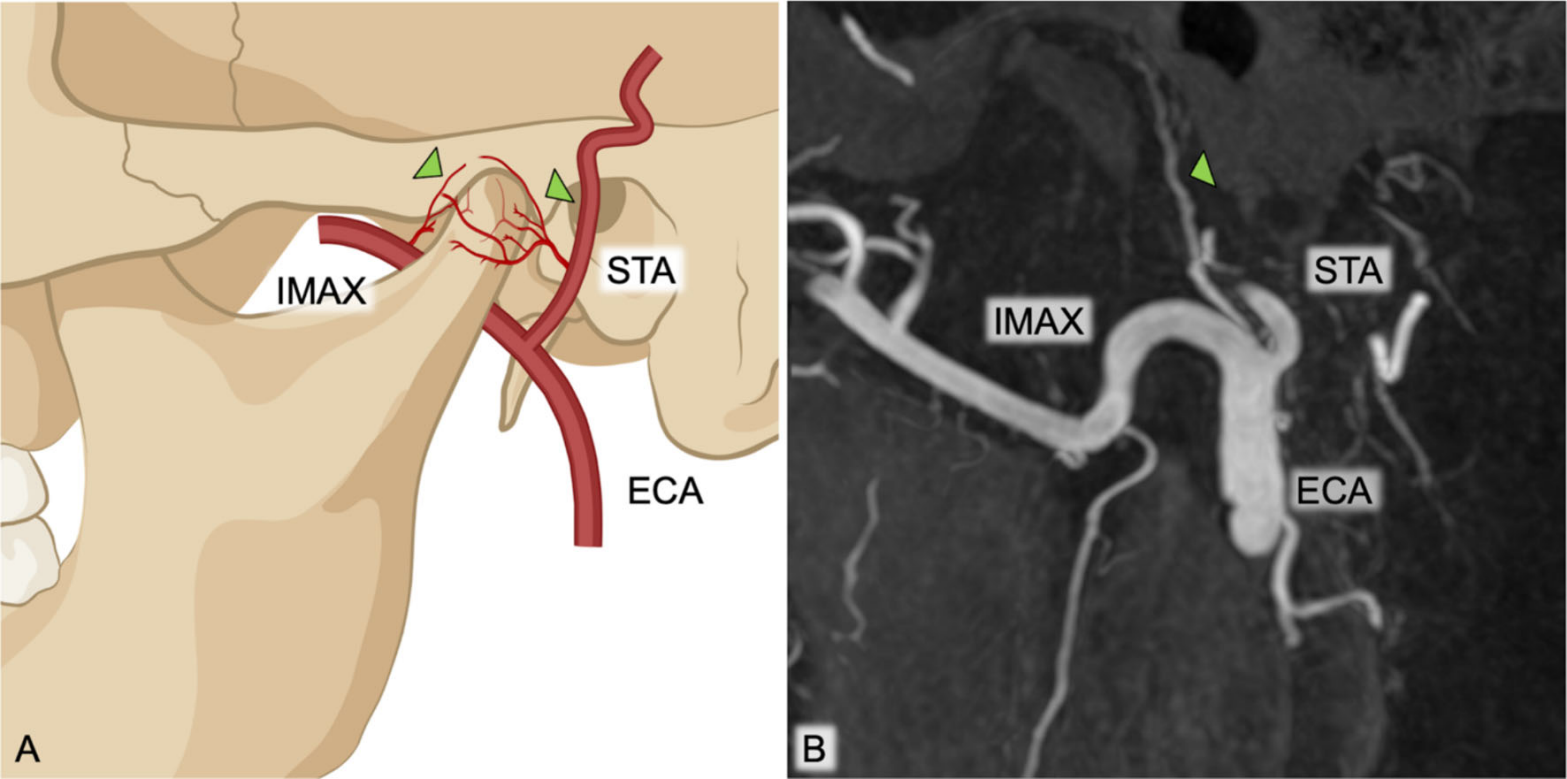
A. Schematic representation and B. MIP-CBCT images of the circumferential blood supply to the TMJ mainly originating from the STA and the IMAX. Green arrows mark joint-feeding arteries, respectively. MIP maximum intensity projection; CBCT cone-beam CT; TMJ temporomandibular joint; STA superficial temporal artery; IMAX internal maxillary artery; ECA external carotid artery

**Fig. 2:**
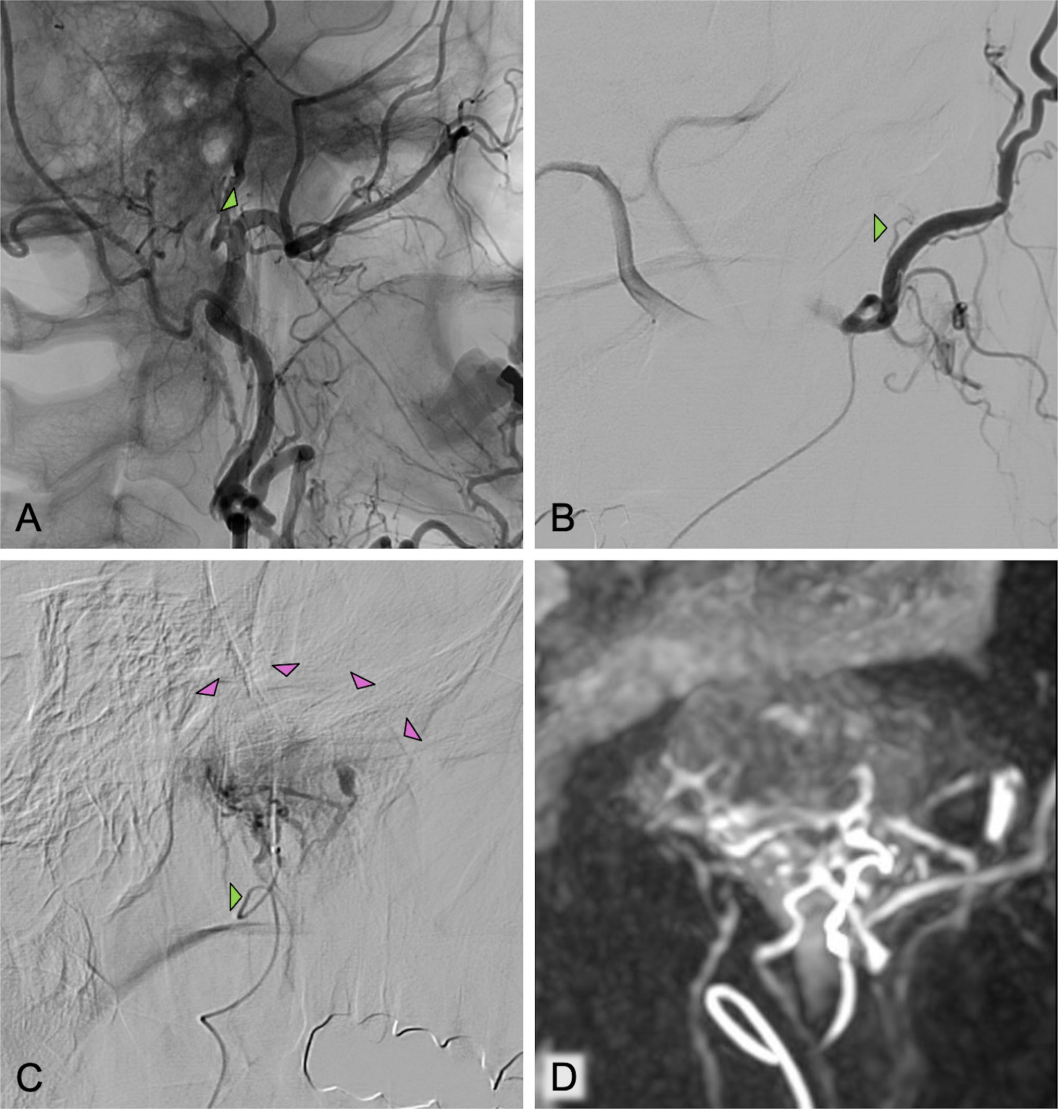
A. Angiogram from the ECA to identify the TMJ-feeding arteries and to evaluate for potential extra- intracranial anastomoses. B. Selective angiogram of the IMAX with an early origin of the dominant TMJ-feeding artery (green arrows, respectively) C. Superselective angiogram of the TMJ-feeding artery revealing the OA-typical hyperemic blush. The purple arrows mark the joint space confirming correct positioning on the angiogram. D. MIP-CBCT reconstruction of the 3D angiogram from the superselective tip position of the microcatheter revealing a net of arteries formed around the TMJ (rete articulare mandibula). TMJ temporomandibular joint; ECA external carotid artery; IMAX internal maxillary artery; MIP maximum intensity projection; CBCT cone-beam CT

Prior to treatment, a contrast-enhanced MRI of the TMJ was performed to confirm synovial enhancement and exclude other TMJ-pathologies. TAE was performed using a biplane angiography system (Azurion 7, Philips Healthcare) by a team that included an interventional radiologist (FF or FC, with 6 and 8 years of angiography experience, respectively) and an interventional neuroradiologist (ES, with 12 years of experience in neuroangiography). Under local anesthesia, retrograde femoral access was established using a 5-Fr introducer sheath (Radiofocus Introducer II; Terumo). A continuously flushed 5-Fr guiding catheter (ENVOY MDP, Johnson & Johnson) was then positioned in the external carotid artery (ECA). Both, digital subtraction angiography as well as 3D rotational angiography with cone-beam CT (CBCT) reconstruction were performed to identify the blood supply to the TMJ and to evaluate potential extra-intracranial anastomoses. Briefly, both superficial temporal artery and internal maxillary artery can connect to the ophthalmic as well as to the internal carotid system, giving rise to the possibility of stroke and blindness due to off-target embolization [10, 11]. Figure 1 shows the circumferential blood supply to the TMJ mainly originating from the proximal part of the superficial temporal artery and the internal maxillary artery. Once the target vessels were identified, superselective microcatheterization was performed using a 0.014" microguidewire (Synchro 14 SELECT, Stryker) and a 1.7 Fr microcatheter (Headway duo, Terumo). To confirm correct positioning of the microcatheter, a second CBCT with maximal magnification and narrow collimation was performed before embolization, as shown in Fig. 2. Superselective embolization was performed using Imipenem/Cilastatin (IPM/CS, 500 mg/500 mg, Merck & Co.). To prepare the embolic agent, one vial of IPM/CS in powder form was diluted in 10 ml of pure contrast medium (Ultravist-370, Bayer Vital). A total of 1–3 ml of the mixture was administered in aliquots of 0.1 ml per injection, continuing until complete stasis was achieved in the target vessel [12]. Following embolization, the status of arterial flow of both the superficial temporal artery and internal maxillary artery was evaluated by an ECA angiogram. All patients were routinely admitted for observation overnight and received an ultrasound examination of the puncture site before discharge the day after TAE.

### Follow-Up and Outcome Measures

TMJ-OA-related symptoms were assessed using the numeric rating scale (NRS), as well as the Oral Health Impact Profile—Temporomandibular Joint (OHIP-TMJ) questionnaire, a specialized tool designed to assess quality of life. During follow-up, patient-reported outcomes were measured at baseline, 6 weeks, and at 3 months intervals. If bilaterally treatment was planned, the follow-up period started after the second treatment. Adverse events were evaluated immediately after the procedure, 24 h postprocedure at patient discharge, and during scheduled follow-up visits, using the Society of Interventional Radiology adverse event classification system [13]. Technical success was defined as the superselective embolization of at least one artery supplying the TMJ.

## Results

Three female patients (age 28, 44 and 72 years) underwent a total of six TAE procedures. Two patients were planned for bilateral treatments spaced four weeks apart. Baseline patient characteristics and prior TMJ-specific treatments are shown in Table 1. In each of the six procedures, the target vessels were identified, and superselectively embolized. Technical success rate was 100%. Clinical follow-up data after 3 months are available for all three patients, one patient reached the 6-months follow-up. The OHIP-TMD score of the three patients decreased from 38, 45 and 45 to 31, 39 and 28, respectively. Over the same time, pain, as reported by the NRS score improved from 9, 10 and 9 to 6, 7 and 5, respectively. No adverse events were observed. One patient (patient II) presented with end-stage TMJ-OA, characterized by bilateral joint dislocation and trismus. TAE was initially planned as a bridging pain management therapy prior to customized joint replacement. Due to pain recurrence after the first treatment, the left side was treated twice, with an interval of eight weeks between procedures. While night pain showed consistent relief after 4 weeks, joint functionality improved only temporarily. Approximately one year after the first TAE, the patient underwent bilateral arthroplasty.

**Table 1:** Patient demographics, procedures and outcomes

	Patient I		Patient II			Patient III
Age	28		44			72
Sex	Female		Female			Female
Previous conservative treatment	- Physical therapy		- Physical therapy			- Physical therapy
	- Splint therapy		- Splint therapy			- Splint therapy
	- Botulinum neurotoxin injection		- Botulinum neurotoxin injection			- Analgesic therapy
	- Analgesic therapy		- Analgesic therapy			
Previous surgical treatment	- Augmentation of the eminence with autologous bone graft		- Arthroscopy-assisted lavage			
	- Arthrocentesis		- Bilateral resection of temporal styloid process			
	- Eminectomy		-Discectomy			
	- Scar release and arthroscopy-assisted lavage					
	- Arthrocentesis					
Sequence of procedures	right	left	left	right	left	right
Technical success	100%	100%	100%	100%	100%	100%
IPM/CS used	2 ml	2 ml	1 ml	2 ml	2 ml	1 ml
Origin of joint-feeding vessel	IMAX	IMAX & STA	STA	IMA	STA	IMAX
OHIP-TMD at Baseline	38			45		45
OHIP-TMD at 4W	29			40		29
OHIP-TMD at 3 M	31			39		28
OHIP-TMD at 6 M	N/A			N/A		28
NRS at Baseline	9			10		9
NRS at 4W	5			8		6
NRS at 3 M	6			7		5
NRS at 6 M	N/A			N/A		5

STA superficial temporal artery; IMAX internal maxillary artery; OHIP-TMJ Oral Health Impact Profile—Temporomandibular Joint; NRS Numeric Rating Scale

## Discussion

This study presents our initial experience with TAE as a minimally-invasive treatment for refractory TMJ-OA. Our findings reported in this small case series highlight the potential of TAE to provide symptom relief for carefully selected TMJ-OA patients with limited treatment options. After a mean follow-up of 13.3 weeks, all patients experienced a remarkable reduction in pain and improved OHIP-TMD scores. However, we also observed that patients with very advanced, end-stage disease, such as patient II in our study, may derive limited functional benefit from TAE. For such cases, TAE may serve as a bridging therapy for pain management before definitive surgical interventions like joint replacement.

One of the primary concerns with embolization procedures in the head and neck region is the risk of non-target embolization, particularly given the complex vascular anatomy of the TMJ and its immediate proximity to the skull base and the brain. The TMJ receives blood supply from multiple arterial sources, including the superficial temporal artery and the internal maxillary artery, both of which have potential anastomoses with intracranial or orbital vasculature 10, 11, 14. Due to anatomic variability as well as safety considerations, we strongly advocate for the use of intraprocedural CBCT to effectively target pathological vessel structures and minimize the risk of non-target embolization.

In conclusion, based on a small patient sample and limited follow-up period, our findings indicate that TAE with IPM/CS could be a technically viable option for managing treatment-resistant chronic pain and joint dysfunction caused by TMJ-OA.

## Abbreviations

TAE: Transarterial Embolization
TMJ: Temporomandibular Joint
OA: Osteoarthritis
MRI: Magnetic Resonance Imaging
ECA: External Carotid Artery
STA: Superficial Temporal Artery
IMAX: Internal Maxillary Artery
MIP: Maximum Intensity Projection
CBCT: Cone-Beam Computed Tomography
IPM/CS: Imipenem/Cilastatin
OHIP-TMD: Oral Health Impact Profile – Temporomandibular Joint
NRS: Numeric Rating Scale
CT: Computed Tomography
