# Correction to: Transcatheter Arterial Embolization as a Treatment for Chronic Pain due to Temporomandibular Joint Osteoarthritis

**DOI:** 10.1007/s00270-025-04053-3

**Published:** 2025-05-16

**Authors:** Florian Nima Fleckenstein, Jan Voss, Christian Doll, Tazio Maleitzke, Tobias Winkler, Eberhard Siebert, Federico Collettini

**Affiliations:** 1 Department of Diagnostic and Interventional Radiology, Charité Universitätsmedizin Berlin, Corporate Member of Freie Universität Berlin and Humboldt-Universität zu Berlin, Berlin, Germany; 2 Berlin Institute of Health at Charité Universitätsmedizin, Berlin, Germany; 3 Department of Oral and Maxillofacial Surgery, Charité Universitätsmedizin Berlin, Corporate Member of Freie Universität Berlin and Humboldt-Universität zu Berlin, Berlin, Germany; 4 Center for Musculoskeletal Surgery, Charité Universitätsmedizin Berlin, Corporate Member of Freie Universität Berlin and Humboldt-Universität zu Berlin, Berlin, Germany; 5 Berlin Institute of Health at Charité Universitätsmedizin Berlin, Julius Wolff Institute, Berlin, Germany; 6 Department of Orthopedic Surgery, Trauma Orthopedic Research Copenhagen Hvidovre (TORCH), Copenhagen University Hospital - Amager and Hvidovre, Hvidovre, Denmark; 7 Department of Clinical Medicine, University of Copenhagen, Copenhagen, Denmark; 8 Institute of Neuroradiology, Charité Universitätsmedizin Berlin, Corporate Member of Freie Universität Berlin and Humboldt-Universität zu Berlin, Berlin, Germany; 9 Department of Radiology, Charité - Universitätsmedizin Berlin, Charité Campus Mitte (CCM),Charitéplatz 1, Berlin, 10117 Germany

**Correction to: Cardiovasc Intervent Radiol** 10.1007/s00270-025-04008-8

In the originally published version of this article, the title was incorrectly rendered. The correct title should read:

"Transcatheter Arterial Embolization as a Treatment for Chronic Pain due to Temporomandibular Joint Osteoarthritis."

This error has been corrected in the online version of the article. The publisher apologizes for the oversight.

The original article has been corrected.

